# Survivability of Deterministic Dynamical Systems

**DOI:** 10.1038/srep29654

**Published:** 2016-07-13

**Authors:** Frank Hellmann, Paul Schultz, Carsten Grabow, Jobst Heitzig, Jürgen Kurths

**Affiliations:** 1 Potsdam Institute for Climate Impact Research, P.O. Box 60 12 03, 14412 Potsdam, Germany; 2 Department of Physics, Humboldt University of Berlin, Newtonstr. 15, 12489 Berlin, Germany; 3 Institute for Complex Systems and Mathematical Biology, University of Aberdeen, Aberdeen AB24 3UE, United Kingdom; 4 Department of Control Theory, Nizhny Novgorod State University, Gagarin Avenue 23, 606950 Nizhny Novgorod, Russia

## Abstract

The notion of a part of phase space containing desired (or allowed) states of a dynamical system is important in a wide range of complex systems research. It has been called the safe operating space, the viability kernel or the sunny region. In this paper we define the notion of survivability: Given a random initial condition, what is the likelihood that the transient behaviour of a deterministic system does not leave a region of desirable states. We demonstrate the utility of this novel stability measure by considering models from climate science, neuronal networks and power grids. We also show that a semi-analytic lower bound for the survivability of linear systems allows a numerically very efficient survivability analysis in realistic models of power grids. Our numerical and semi-analytic work underlines that the type of stability measured by survivability is not captured by common asymptotic stability measures.

In almost all dynamical systems applicable to the real world, the stability of the system’s stationary states (periodic orbits, chaotic attractors, etc.) is of key interest, because perturbations are never truly absent and initial data is never exactly determined. Nevertheless, the asymptotic stability of the system’s attractors ensures that we can still extract sensible long-term information from our dynamical models.

Complementary to the notion of stability, one can analyse whether the system will remain in a desirable regime^1^. This becomes important when a model represents a system that we have influence on, either because we engineer its fundamental behaviour, or because there are management options. We often want to design the dynamics, or our interventions, such as to more easily keep the system in such a desired state. Note that the desirable region not necessarily contains a stationary state.

For the traditional notion of asymptotic stability against small perturbations, the key mathematical concept is the analysis of the linearised dynamics, in particular by means of the Lyapunov exponent or master stability function^2^,^3^.

Real-world systems typically are multistable^4^,^5^,^6^. They have more than one stable attractor^7^, and thus potentially exhibit a wide range of different asymptotic behaviours. The key question then becomes from which initial state which attractor is reached, i.e., to determine the basin of attraction of an attractor. Most work so far focused on the geometry of the basin of attraction^8^ of desirable attractors, e.g. by finding Lyapunov functions^9^,^10^,^11^.

A recent idea that has been found to be useful is to study a more elementary property, i.e. not which states go to an attractor, but just how many. This quantity, the volume of the basin of attraction of a given attractor, can then be interpreted as the stability of the system in the face of a random, non-small perturbation. It quantifies the probability that the typically non-linear response to such a perturbation will lead the system to a different, undesirable attractor. This probability is called the *basin stability* (*S*_*B*_) of an attractor^12^. This is important for a number of applications where relevant system deviations are typically not small, for example in neuro science, system Earth or power grids.

One of the key appealing features of *S*_*B*_ is that, by studying just the volume rather than the shape of the basin of attraction, it becomes numerically tractable to analyse even very high-dimensional systems. It was also shown that the information revealed by the volume of the basin genuinely complements the information provided by the Lyapunov exponents of the system^12^.

There are, however, two major drawbacks when estimating *S*_*B*_. On the one hand, the measure relies on identifying the asymptotic behaviour of a system, which might be difficult to detect, typically requires prior knowledge about the attractor’s nature, and is only meaningful in multistable systems. On the other hand, a *S*_*B*_ estimation is insensitive to undesired transient behaviour of the system, i.e. if the trajectory visits an undesired part of the phase space where the system would take damage that is not modelled explicitly. To detect this type of dangerous transients, a new, complementary measure is required.

In this paper we introduce a new stability-related measure, the *survivability S*(*t*) of a dynamical system. This is the fraction of initial system states (i.e. arising from an initial large perturbation) giving rise to evolutions that stay within a desirable regime up to a given time *t*. The set of these initial conditions is called *basin of survival*.

More formally, call the phase space of our system *X*, and a chosen desirable region 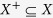. The finite-time basin of survival 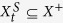 is defined as the set of initial conditions in *X* for which the entire trajectory over the interval [0, *t*] lies in *X*^+^. We choose a probability measure *μ* of initial conditions, reflecting our knowledge of the nature of perturbations we wish to study. Accordingly, the *finite-time survivability* is defined as


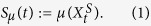


The total survivability then is the infinite-time limit of *S*_*μ*_(*t*). This can naturally be decomposed into the probability that the initial perturbation is survived, and that the following trajectory stays save:


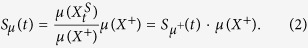


with 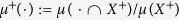. Now *μ*(*X*^+^) does not depend on the dynamics but only on the desirable region and the perturbations, i.e. it is a constant for given *X*^+^. The conditional survivability 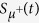 captures the interplay of dynamics, desirable region and perturbations; it has a natural interpretation as the conditional probability of a system to survive random, large perturbations that do not kill it immediately.

Assuming a uniform distribution of perturbations, the measure *μ* is proportional to the volume Vol. The resulting conditional survivability is our main object of study in what follows. We will call this finite-time survivability of a dynamical system:


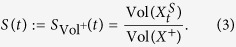


We are also interested in initial perturbations that only occur in a particular region of phase space. Thus, we want to study uniform perturbations in a subset *C* ⊂ *X*. The conditional survivability *S*^*C*^(*t*) can then simply be defined with respect to the measure Vol^*C*^(⋅) = Vol(⋅ ∩ *C*)/Vol(*C*):


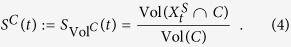


An important example of such a conditionial survivability is the single node survivability for networked systems. There we condition on the phase space at a single node, thereby isolating the impact of local perturbations on the whole system. A mathematically precise discussion will follow in the power grid example in the results section and the supplementary information (SI).

To further illustrate this definition, consider a simple example: A penguin wishing to ski down a mountain *X* going the fastest route possible in Fig. 1. The system is multistable as the penguin might end up in the goal or the valley. However, if the penguin goes over the cliff it will almost certainly slide the rest of the way to the goal on its back. The state of the penguin is not explicitly modelled by our (potential) landscape. We take this into account by declaring the parts of the cliff our penguin can not ski safely as an undesirable region. Further, if the penguin wishes to continue skiing, the valley might or might not be undesirable as well. Depending on these choices, different starting points can be in the basin of survival. If the goal is the only desirable attractor, the basin of survival lies in its basin of attraction, but if the valley is OK, too, this is not the case, and the asymptotic structure plays no role.

As opposed to *S*_*B*_ or a linear(-ised) analysis based on Lyapunov exponents, the survivability is concerned not just with the asymptotic behaviour of the system, but depends strongly on the transient dynamics. As opposed to *S*_*B*_ it is applicable in unstable, mono-stable, or multistable, linear or non-linear systems.

The application of the survivability concept is especially appropriate when interventions happen at the same time scale as the system dynamics, or when entering an undesirable region is deadly.

A key insight is that evaluating survivability becomes amenable to Monte Carlo integration. This is due to focusing on the probability that the trajectory following a perturbation violates the boundary rather than trying to find the actual sets of phase space from which a trajectory survives. Hence, a survivability analysis, just as *S*_*B*_, is applicable to very high-dimensional systems. In fact, the situation is more favourable than in the case of *S*_*B*_, as the entire curve *S*(*t*) can be evaluated at a computational cost not exceeding that of *S*_*B*_, while potentially revealing much more information.

This sets survivability apart from formally similar approaches, e.g. in control theory^13^,^14^. Their precise relationship to survivability is discussed in detail in the *Methods* section.

For linear systems with a polyhedral desirable region, we derive a closed form lower bound on the *infinite-time survivability S*_∞_ : = *S*(*t* → ∞) as well as a semi-analytic, stronger bound that becomes exact in the case of vanishing dissipation. These bounds reveal that the survivability of linear systems depends strongly on the eigenvectors of the linear dynamics, rather than just the eigenvalues. The semi-analytic bound eliminates the need to simulate the system trajectory opening survivability up to a wide range of applications for which numerically estimating the full dynamics is not feasible.

## Results

To demonstrate the diverse applicability of our survivability concept we apply it to three paradigmatic model systems. A two-dimensional model of carbon stock dynamics, a system of integrate-and-fire neurons and a high-dimensional network model of the power grid.

These systems were chosen to cover a wide range of types of systems. The carbon cycle model has one or two attractors, depending on the parameter regime, and some transients are deadly. The neurons are mono-stable but exhibit transient chaos^15^,^16^,^17^,^18^,^19^. Finally the power grid model is high-dimensional, non-linear and multistable. However, the acceptable operating regime is close to a certain class of fixed points, thus the linearised behaviour near these fixed points is of great practical importance.

In all three systems there are externalities which are not or cannot be modelled explicitly. Namely, the influence of dramatic climate changes on society, external stimuli for a network of neurons and frequency control mechanisms in the power grid. We will see that survivability accurately captures the interplay of externalities with the intrinsic dynamics.

### Carbon cycle model by Anderies *et al*

We begin by applying survivability to a two-dimensional carbon cycle model from climate science which has been recently introduced^20^. This is a conceptual model with the aim to reproduce the non-linear dynamics of the carbon cycle in the Earth system. The boundaries of the *survival region* are closely related to the concept of planetary boundaries^21^. This system exhibits both the property that the undesirable states are deadly and that in some parameter regimes there is only a single stable attractor of the asymptotic dynamics.

The model equations for the atmospheric (*c*_*a*_), marine (*c*_*m*_) and terrestrial (*c*_*t*_) carbon stocks are given by


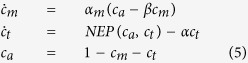


where *α*_*m*_ denotes the atmosphere-ocean diffusion coefficient, *β* the carbon-solubility in sea water factor, *α* the human terrestrial carbon off-take rate and *NEP*(*c*_*a*_, *c*_*t*_) the net ecosystem production, a complex non-linear relationship between the atmospheric and terrestrial carbon stocks (see Anderies *et al.*^20^ for further details). Note that the total amount of carbon is kept constant, leaving us with the marine (*c*_*m*_) and terrestrial (*c*_*t*_) carbon stocks as independent variables.

Part of the phase space *X* of the model are states with virtually no terrestrial carbon, referred to as *desert states*. While the model can recover from such states and eventually reach high terrestrial carbon states again, entering a desert state would lead to the collapse of human civilisation and thus, tragically, our model would no longer be valid after entering this regime. Hence, we define the set of desirable states *X*^+^ as the complement of the desert states plus a safety margin *m*:


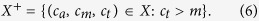


The safety margin should at no time, during the transient or asymptotic behaviour, be crossed. The finite-time basin of survival, here introduced as 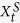, is then given by


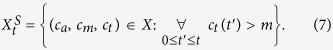


**Figure 2:**
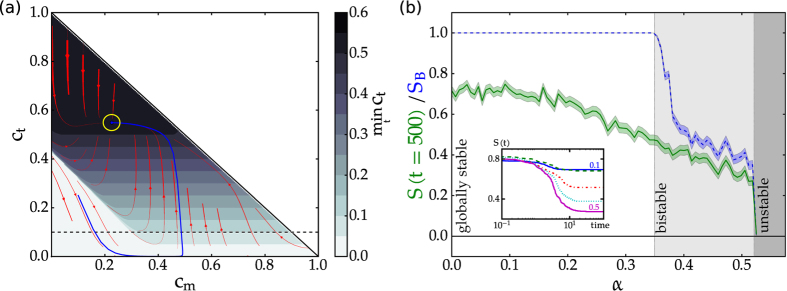
(**a**) Phase portrait of Anderies’ model (Eq. 5, *α* = 0.1). We choose initial terrestrial (*c*_*t*_) and marine (*c*_*m*_) carbon stocks, the colour scale then indicates the minimum of *c*_*t*_ over the whole time evolution commencing from a point. An example trajectory with a long excursion to the desert state (*c*_*t*_ < *m*) is plotted in blue and ends at the attractor which is circled in yellow, the stream plot indicates the vector field of the right-hand-side (cf. Eq. 5). The dashed black line indicates the value of the safety margin *m* = 0.1. (**b**) Bifurcations in the carbon cycle model. Basin stability (*S*_*B*_, blue) and finite-time survivability (*S*(*t* = 500), green) estimates for different values of the terrestrial human carbon off-take *α*. For the survivability estimation we assumed a safety margin *m* = 0.1. The shading around the curves indicates one standard error, the background colour indicates the different dynamical regimes. In the inset, we give survivability curves for five selected values of *α*, i.e. *α* ∈ {0.1, 0.2, 0.3, 0.4, 0.5} from top to bottom as indicated.

A phase plane analysis for this model is illustrated in Fig. 2(a). Of special importance here are those trajectories (exemplified by the blue trajectory in Fig. 2(a)) that first cross the safety margin, i.e. are not desirable due to the very low terrestrial carbon stocks *c*_*t*_, but eventually will return to the desirable region *X*^+^. These trajectories are counted for the *S*_*B*_ estimation, since they eventually approach the attractor, but are disregarded for the survivability, since they cross the safety margin during the transient period.

By varying the human carbon off-take *α* in Eq. 5, the system undergoes a bifurcation changing the number of attractors (around *α* = 0.35) as illustrated in Fig. 2(b). The main picture shows the asymptotic survivability, the inset contains the survivability curves for different values of *α*. We see that the survivability drops to the asymptotic plateau at around the same time. Thus, if a trajectory eventually leaves the desirable regime, the time it takes until it does so is not strongly affected by *α*.

The bifurcation, which is known to be a saddle-node bifurcation^20^, has a drastic impact on the *S*_*B*_ estimation, the survivability only changes marginally in this interval. On the other hand, the behaviour in the interval *α* ∈ [0; 0.35] shows how the *S*_*B*_ estimation becomes insensitive to system changes if the multistability is lost, i.e. if there is only a single attractor (in this case with non-zero *c*_*t*_). The crucial question whether trajectories stay in a desired regime is thus not captured by the *S*_*B*_ measure, but can be answered with the survivability concept. Note that in this case and in what follows we estimate a finite-time survivability for the entire simulated time evolution of the system. Given that the asymptotic behaviour sets in earlier than the simulation ends, this is a good estimate for the infinite-time survivability.

It was argued^12^ that *S*_*B*_ can also serve as a better early warning indicator of approaching tipping points than other measures. Here we see that a survivability estimation mirrors the trend in the system’s behaviour, i.e. how the set of surviving states depends on system parameters, while *S*_*B*_ remains fixed at its plateau value. Hence, survivability can serve as a complementary, and in some scenarios better early warning sign than *S*_*B*_.

### Network of integrate-and-fire oscillators

In the case of transient chaos^15^,^16^,^17^,^18^ there are long, interesting transients but potentially just a single global attractor. As an example, we consider a network of *N* integrate-and-fire neurons^22^,^23^,^24^,^25^. They exhibit long-term chaotic transients, but asymptotically have a global periodic attractor where the neurons are in a state of phase-synchronisation. Considering the synchronised state as undesirable, the integrate-and-fire neurons are an example of a system in which neither asymptotic nor basin stability are informative.

Modelling external stimuli as essentially randomly resetting the phases of stimulated neurons, the survivability *S*(*t*) here carries the interpretation of the probability that the system will not fall into a synchronised state in between stimuli, spaced apart at interval *t*. Such synchronised states model epileptic seizures and are thus undesired.

Concretely we study the convergence from arbitrary initial conditions to periodic orbit attractors, in which several synchronised groups of oscillators (clusters) coexist^26^. In the network every oscillator *j* = 1…*N* is connected to another oscillator *i* ≠ *j* by a directed link with probability *p*. A phase variable *ϕ*_*j*_(*t*) ∈ [0, 1] specifies the state of each oscillator *j* at time *t*. The free dynamics of an oscillator *j* is given by


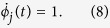


The oscillators interact on a directed graph by sending pulses when they reach the threshold *ϕ*_*j*_ = 1. After a delay time *τ* this pulse induces a phase jump (indicated by differentiating the left and right limit of *t* as *t*^+^ and *t*^−^) in the receiving oscillator *i*:


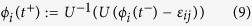


for a potential *U* and coupling strength *ε*_*ij*_ (For more details cf. the SI).

**Figure 3:**
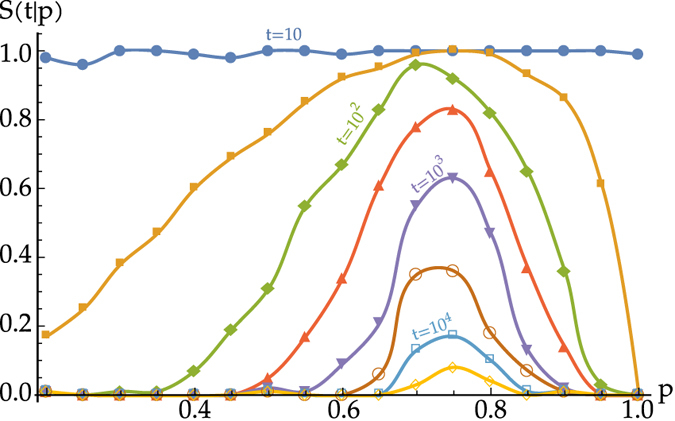
Survivability curves for networks of integrate-and-fire oscillators. Finite-time survivability *S*(*t*|*p*) for given survival times *t* vs. the network parameter *p*. For each value of *p* we average over an ensemble of 100 network realisations, each with initial conditions drawn at random from the full state space.

The survivability *S*(*t*|*p*) for a directed network of *N* = 16 pulse-coupled oscillators in dependence on the average connectivity *p* is illustrated in Fig. 3. For each value of *p* we create an ensemble of 100 network realisations. The randomly chosen initial phase vectors for each realisation are distributed uniformly in [0, 1]^*N*^.

All different network realisations with their associated initial conditions eventually lead to a fully synchronous state. However, our concept of survivability reveals the highly non-linear, non-monotonic dependence on the network connectivity *p*. While the survivability of transient dynamic states is small for networks with low and high connectivity values *p*, it becomes very large for intermediate connectivities, even for only weakly diluted networks (Fig. 3). The finite-time survivability reveals a new, collective time scale that is much larger than the natural period, 1, of an individual oscillator and the delay time, *τ*, of the interactions.

These long, irregular transients are the main property of interest for the system, motivating their study in ref. 26. The dependence of the average lifetime of the transient chaotic trajectories on *p* was already studied ibidem. In this example, survivability reveals the same dynamical information as previous studies. Note that this is due to the specific choice of desirable region as the non-periodic parts of state space. Generally, there is no direct relationship between survivability and transient lengths, the fact that the desirable region can be chosen such that survivability reveals the quantity of interest for this system in a natural way speaks for its universality.

Survivability again is a natural and informative stability measure of this system, however, this time not against perturbations, but against getting trapped in an undesired corner of phase space.

### Power grids

Power grids are subject to a variety of failures and perturbations and there are numerous studies concerning asymptotic stability analysis, e.g. refs 27 and 28, and recent approaches to an *S*_*B*_ assessment^29^,^30^. However, contrary to common model assumptions, the dynamical system does usually not evolve freely after a perturbation. If the system does not return to a stable operating state after a typical time span of a few seconds or if predefined thresholds are exceeded, control mechanisms that would require independent modelling are triggered.

The long-term behaviour and stability of the system is thus a question for control theory rather than just dynamics. Conversely, the transient dynamics, and the question whether there is a temporary amplification of perturbations, is critical to whether the control has to be activated at all, or the system is explicitly resilient to such perturbations. Hence, the power grid is an example where the undesirable region is deadly and management options operate at the system dynamics time scale.

The effective network model of the power grid^31^,^32^ is the current standard baseline model for the frequency dynamics of power grids. It is known as the *swing equation* or the second-order Kuramoto model, and is used for short-term frequency stability studies in power grids. The various ways in which a power grid can be modelled using the swing equation are discussed in ref. 32 and limits to its applicability are discussed, for example, in refs 33 and 34.

The dynamical system modelling *N* generators’ instantaneous phases *ϕ*_*i*_ and frequency deviations *ω*_*i*_ from the grid’s rated frequency is given as


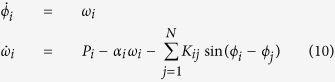


with *P*_*i*_ being the net input power/consumption, *α*_*i*_ the electro-mechanical damping at node *i* and *K*_*ij*_ as the capacity of the link *i* – *j*. Here we choose *P*_*i*_ = 1 for net generators, *P*_*i*_ = −1 for net consumers, and a uniform distribution of *α*_*i*_ = *α* = 0.1. We choose the nonzero *K*_*ij*_ uniformly equal to 6, corresponding to an average transmission line length of about 200 km.

A stable operating state of the power grid is a fixed point of the dynamics with no frequency deviation, 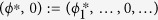. Conversely, limit cycle solutions (frequency oscillations) need to be prevented in order to avoid the tripping of generators. Frequency deviations are usually kept very small in large real power grids, with typical thresholds of ±0.2 Hz^35^ which corresponds to a phase velocity deviation of |*ω*| ≈ 0.25 in our units. Smaller island grids have considerably larger fluctuations. As an illustrative extreme case we will consider up to 20 times larger fluctuations. For *S*_*B*_ assessments, the reaction of the system to much larger deviations was also taken into account.

We will study the *single-node basin of survival*, i.e., the conditional basin of survival in the sense of Eq. 4, conditioned on initial perturbations that occur locally at a single node *n*, starting from a stable operating state. The space we wish to condition on is then the direct product of the stable operating state at all nodes except node *n* and the full state space of the node dynamics at *n*:


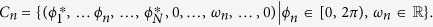


The desirable region being defined as ∀*i* : |*ω*_*i*_| < 5, which, as explained above, is chosen to mirror realistic constraints. Concretely, this means that we construct initial conditions by setting *ϕ*_*i*_ and *ω*_*i*_ to the value of the fixed point 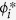 and 0, for all nodes other than the node *n* we are studying, and to a random phase in [−*π*; *π*] as well as a random frequency deviation in [−5; 5] for the node *n*. Then we simulate the system up to *t* = 100 and observe whether (and if, when) any of the frequency deviations *ω*_*i*_ leave the desirable region. In this way we sample 300 trajectories to estimate 
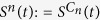
. This leads to a standard error of less than 0.03 for *S*^*n*^(*t*) = 0.5 in the worst case (see Methods section). We evaluate the survivability up to 100 in simulation time (18 s in real time), at which point a steady state has typically been established, and the asymptotic value of the survivability is reached.

While *S*_*B*_ captures the overall ability of the system to avoid permanent frequency oscillations, it does not directly capture the stability of the system against large perturbations. Instead, as discussed above, it is the ability of the system to keep perturbations under fixed frequency thresholds which is crucial. We will study this form of stability using both numerical simulations and the analytic approximations we have derived. The former will allow us to compare the survivability of the system to its *S*_*B*_, the latter to assess the accuracy of our bounds.

We now turn to the question whether the semi-analytic bounds on the dynamics linearised around the fixed point can accurately mirror the single-node survivability *S*^*n*^(*t* = 100).

Defining *ϕ* : = (*ϕ*_1_, …, *ϕ*_*N*_)^*T*^, *ω* = (*ω*_1_, …, *ω*_*N*_)^*T*^ and *α* : = *diag*(*α*_*i*_), the linearised dynamics is given by


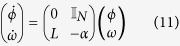


where the lower left block (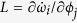) can be identified with the network’s Laplacian matrix (at the fixed point (ϕ^*^, 0)) given by


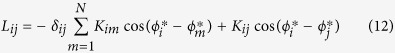


The Jacobian has two real eigenvalues, *λ*_1_ = 0 and *λ*_2_ = −*α*, corresponding to the eigenvectors (*ϕ*, *ω*)_1_ = (1, …, 0, …) and (*ϕ*, *ω*)_2_ = (−1/*α*, …, 1, …). The first eigenvalue, *λ*_1_ and the corresponding eigenvector show the linearised version of the rotational symmetry of the system under shifting all elements of *ϕ* by the same amount *ϕ*_*s*_: 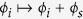. The second corresponds to a homogeneous shift of all oscillator’s frequencies, which does not affect the phase differences, and decays exponentially due to the damping term. The remaining part of the spectrum consists of *N* − 1 pairs of complex conjugated eigenvalues.

**Figure 4:**
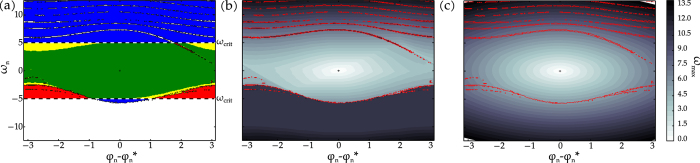
Single-node phase space of a consumer in the Scandinavian grid. (**a**) We plot the initial frequency deviation *ω*_*n*_ vs. the phase difference to the fixed point at node *n*, visualising the definition of the following areas using the simulation results from (**b**). The central green area resembles the infinite-time basin of survival, while the yellow and red areas contain finite-time surviving states. The union of the blue, yellow and green regions resembles the synchronous state’s basin of attraction, while trajectories starting in the white or red regions approach different attractors. The frequency threshold is chosen as *ω*_*crit*._ = ±5 and initial conditions correspond to perturbations at a single consumer node of the network. (**b**) Simulated maximum frequency deviations *ω*_*max*_ along all dimensions, measured over the time evolution of the system for initial conditions that correspond to perturbations at node *n* of the network. For comparison with (**a**), we give the numerically estimated basin of attraction’s boundaries in red. (**c**) Corresponding analytic upper bound for the maximum frequency deviation (cf. Eq. 16) for the linear approximation.

The basin of attraction in the conditional subspace *C*_*n*_ of this system is illustrated in Fig. 4(a). Concerning survivability, there is a subdivision in three different sets. The desirable region contains infinite- (central green region) and finite-time surviving states (yellow and red regions in the band). Trajectories commencing from the remaining states within the basin of attraction (blue region) eventually reach the attractor asymptotically. Note that there are also finite-time surviving states outside the basin of attraction (red region). A large part of the single-node basin of attraction is centred around the fixed point (ϕ^*^, 0). Within this region we expect the linear approximation to provide a lot of information on the system.

Regarding survivability, Fig. 4(b) shows that the frequency deviations inside the basin of attraction do indeed become large. The shape of the level lines of the frequency deviations corresponds to the basins of survival for different frequency constraints.

Figure 4(c) shows the bound for the frequency deviation of the linearised dynamics calculated according to Eq. 16. This shows a good qualitative agreement with the actually simulated frequency deviations as long as the deviations remain close to the fixed point, e.g. in the range of realistically allowed perturbations (see above). Still, the impact of the non-linearity (e.g. multistability is not captured) on the system becomes apparent, especially further away from the fixed point.

**Figure 5:**
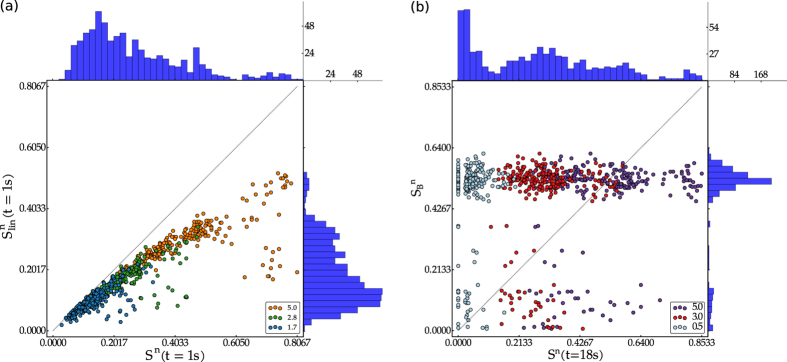
Simulated vs. approximated single-node survivability for the Scandinavian grid. (**a**) Scatter plot of the simulated *S*^*n*^(*t*) vs. approximated single-node survivability 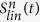 (cf. Eq. 16) estimated for all nodes in the Scandinavian power grid (*ω*_*crit*._ is indicated in the legend). The corresponding distributions are given on the sides. (**b**) Single-node basin stability vs. single-node survivability for the Scandinavian grid. Scatter plot of the single-node basin stability 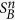 vs. single-node survivability *S*^*n*^(*t* = 100) (*ω*_*crit*._ is indicated in the legend) estimated for all nodes in the Scandinavian power grid. The corresponding distributions are given on the sides. Note that we have chosen the initial region *X*_0_ for single-node basin stability with |*ω*| < 100, the same region as in ref. 29.

Indeed Fig. 5(a) shows that there is a high correlation between the lower bound of the survivability of the linear system 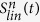 calculated according to Eq. 16 (see *Methods section*) and the actual survivability *S*^*n*^(*t*) at the majority of nodes for realistic values of frequency deviations. What exactly gives rise to the outliers far below the diagonal will require further study. It is important to emphasise that the computational cost of calculating the bounds on the maximum frequency deviation for a sample of initial conditions is many orders of magnitude lower than the numerical estimate of the survivability via simulations of the actual time evolution. For a realistic network size of several hundred nodes, the approximate calculations can be performed on a laptop computer in less than a minute, whereas the numerical survivability estimation took several hours on 200 nodes of a computing cluster.

Figure 5(b) shows 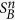 as well as the single-node survivability of nodes in the Scandinavian power grid. We see that there is no significant correlation between the two quantities. This proves the point that the asymptotic behaviour of the system is not a strong indicator of the transient behaviour, at least in the case of power grids. The information we obtain from the survivability analysis is genuinely new information.

The Scandinavian power grid^29^ consists of *N* = 236 nodes and 320 links, corresponding to a mean degree of 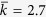. Hence, it has a sparse network topology with only a few neighbours per node on average, which is typical for power grids in general, independent from the number of nodes^36^. The same holds for our second data set, the UK high-voltage transmission grid, which consists of *N* = 120 nodes and 165 links, corresponding to a mean degree of 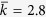.

**Figure 6:**
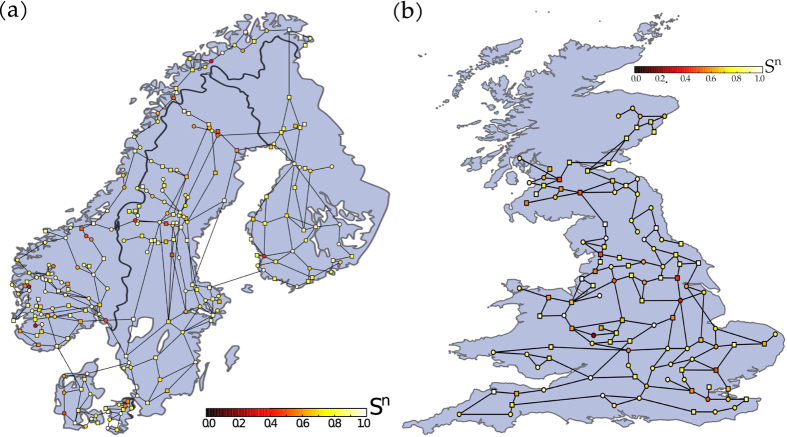
Scandinavian power grid. (**a**) The nodes’ colouring indicates the respective single-node survivability estimate *S*^*n*^(*t* = 1*s*) in the Scandinavian power grid. The frequency threshold is chosen as *ω*_*crit*._ = ±10. We randomly selected a dispatch scenario, circular nodes are net generators, squares are net consumers. The map of Scandinavia has been modified from https://commons.wikimedia.org/wiki/File:Scandinavia.svg, which is licensed under the Attribution-Share-Alike 3.0 Unported license. The license terms can be found on the following link: https://creativecommons.org/licenses/by-sa/3.0/. (**b**) UK power grid. Single-node survivability estimate *S*^*n*^(*t* = 1*s*) of the UK power grid. Details analogous to (a). The map of Great Britain has been modified from https://commons.wikimedia.org/wiki/File:England,_Scotland_and_Wales_within_the_UK_and_Europe.svg, which is licensed under the Attribution-Share-Alike 3.0 Unported license. The license terms can be found on the following link: https://creativecommons.org/licenses/by-sa/3.0/.

In Fig. 6(a,b) we show the geographically embedded Scandinavian and UK power grid. The colour of each node corresponds to the single-node conditional survivability *S*^*n*^(*t* = 1*s*). Different nodes exhibit starkly different survivability to perturbations. We find that at a threshold of |*ω*_*crit*_| = 10, for both of these realistic power grid topologies, there are a few nodes that are particularly vulnerable to perturbations. This means a perturbation at these nodes is very likely to be amplified temporarily by the overall grid dynamics. What exactly leads to this vulnerability, and how to characterise it in terms of grid parameters and topology is a question for future work.

Finally, we also found that the survivability in this system asymptotes very quickly. Simulating just the first second of the power grid is typically sufficient, the so-called “first swing” following a disturbance mainly determines the overall frequency deviation.

Let us summarise the key points from applying survivability to power grids:

- For realistic small deviations, the upper bound applied to the linear approximation provides an excellent picture of the infinite-time basin of survival. The fact that the bulk of nodes shows a high correlation at large perturbations indicates that *S*^*n*^ can still be determined from the approximation in this case.
- For the given dynamics, the survivability very quickly reaches its asymptotic value. We expect this to be a fairly generic phenomenon if we are dealing with damped systems near a stable fixed point.
- Conditioning the survivability on regions of phase space with special meaning, like perturbations at a single node, allows us to reveal a large amount of non-obvious structural information on a networked system. Further work is needed to understand what gives rise to the revealed structure in realistic power grids.

## Discussion

Survivability is a novel stability concept complementary to basin stability *S*_*B*_ and linear methods of asymptotic stability analysis. It applies to linear and non-linear systems, in the absence and presence of multi-stability. It focuses on transient rather than asymptotic behaviour, and incorporates exogenous information via assuming a desirable region for the system dynamics. Further, survivability can be estimated numerically at low computational costs, comparable to or even lower than for estimating *S*_*B*_.

For linear systems we provide easy to evaluate analytic and semi-analytic expressions for lower bounds of the survivability, with a trade-off between the quality of the bound and numerical cost for evaluating the analytic expression. These reduce the need to simulate the system, yielding further dramatic improvements in computational cost.

The bounds we find demonstrate that the survivability depends crucially on the eigenvectors of the linear dynamics, rather than the eigenvalues (see discussion in the *Methods* section). It is an effective measure of the interaction between external constraints and the geometry of the dynamics in its phase space. The fact that the bound is tight exactly when the analysis of asymptotic stability using the eigenvalues of the linearised system fails shows that the survivability is genuinely complementary to eigenvalue-based stability concepts.

To explore this measure in practice, we analyse three conceptual examples.

### Carbon Cycle

We observe that survivability accurately exhibits the presence of dangerous transient behaviour in the model, something that *S*_*B*_ can not detect. The almost monotonous decrease towards the first tipping point, opposed to the discontinuous *S*_*B*_ curve, shows the potential to derive an early warning scheme from an observation of these measures for certain kinds of bifurcations. Just as for *S*_*B*_, the problem of evaluating the survivability from data remains a challenge for future work.

### Neuronal Networks

Here, the transients do not arise from perturbations constructed as deviations around a desirable attractor, but they are randomly chosen from the whole compact phase space. Rather, the main interest lies on the transients themselves. Survivability reveals the same qualitative dependence of the dynamical behaviour on the underlying network topology as the average length of the transient^26^. Beyond that, considering *S*(*t*) at fixed *t* as a function of the underlying topological parameters enables us to look in more detail into the relationship between function and structure of pulse-coupled oscillator networks. In contrast to the average length of the transients, the survivability also has a direct conceptual interpretation as the probability of the system remaining in the interesting transient regime. Thus it captures the appropriate notion of stability of transient chaos against the global attractor.

### Power Grid

In this example we can see in detail the interplay between the semi-analytic bounds that we developed and the fully non-linear system. We demonstrate that survivability under realistic constraints captures information about the system not contained in the *S*_*B*_ estimate. We also demonstrate that the semi-analytic lower bounds, are strongly correlated with the simulations of the non-linear dynamics. Thus they contain much of the relevant information about the system. In strategic power grid development studies, this fact becomes particularly important as computational power is often at a considerable constraint, due to the need to simulate a wide range of divergent future scenarios of the energy transition. Dynamical properties outside of quasi-stationary calculations can only be taken into account if efficient estimators exist, since it is not feasible to run simulations. Thus our lower bounds, which eliminate the need for such simulations, potentially enable a more systematic way to investigate the impacts of the energy transition. In particular, the influence of changing topologies and different distributions of dynamical parameters on the dynamics of the power grid become computationally accessible. For the application to power grids, there are many more operational conditions on the system’s behaviour that we do not consider here. While not all of them are as amenable to analytic considerations as the frequency deviation, we anticipate that it will still be possible to find cheap analytic boundaries for them. The reason that we could calculate the lower bounds so easily is that the phase space geometry is encoded in an efficient way in the eigenvectors. This aspect will carry over to many other, more complicated exogenous boundaries.

We thus have seen that the notion of survivability is general and powerful enough to capture the interplay between externalities and the intrinsic dynamics in three vastly different examples. In particular the last example demonstrated both the utility of single node survivability, revealing structural weaknesses and strengths of realistic power grid topologies, as well as of our semi-analytic bounds, reducing computational efforts dramatically.

The work presented here thus opens up a plethora of new avenues of research. On the theoretical side, the existence of a closed form lower bound on the survivability of a linear system opens the door to study the survivability as a function of the network topology and system parameters analytically, especially for the optimisation of these parameters to increase the system’s survivability. The lower bounds presented here can certainly be improved by taking the more detailed geometry of the trajectories of the linear system into account. It will also be important to extend them to the types of bounds we have in more realistic power grid models.

## Methods

### Numerically estimating survivability

One advantage shared by survivability and^12^ is that they can be efficiently estimated by randomly sampling starting conditions. A trajectory either survives or not, therefore we can regard the sampling as a Bernoulli experiment with probability given by *S*(*t*), hence the standard error (*SE*) of the probability estimator of a trial with *N* draws is simply


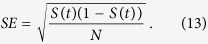


As a crucial consequence, the standard error of a survivability estimation does not depend on the dimensionality of the system. Further, the condition that a trajectory has left *X*^+^ tends to be easier to evaluate in practice than whether the trajectory is asymptotically approaching a fixed point. Furthermore, in numerical simulations, an integration might be stopped once *X*^+^ has been left.

### Analytic results for linear systems

An important analytically tractable case is the total survivability *S*_∞_ for a linear dynamic in 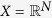, the Lebesgue measure 
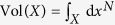
, and a polyhedral desirable region given by *m* linear conditions *y*_*k*_ ⋅ *x*(*t*) < 1 for a set of vectors *y*_*k*_, *k* = 1…*m* in 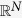. In this case we can give a lower bound on 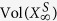 that is easy to evaluate.

In this section we briefly give the results necessary for the applications in the results section on power grids. There we demonstrate that the semi-analytic bound captures the survivability of the system quite accurately in practical examples. In the SI we show detailed derivations, as well as further analytic results.

Consider a system of linear ordinary differential equations


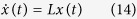


with 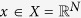 and 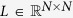 with all eigenvalues having non-positive real parts. In general, *L* has a complex spectrum. The eigenvectors *v*_*j*_ of the complex eigenvalues are real or come in complex conjugate pairs, from which we pick one eigenvector each. We then define the *N* × *N* matrix 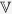 by stacking the eigenvectors, or their real and imaginary parts respectively, against each other as column vectors:


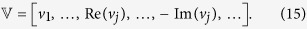


This allows us to translate initial conditions into the eigenvector basis by setting 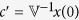, and combining 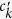 into complex numbers as appropriate 
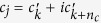
, where *n*_*c*_ is the number of complex eigenvalues. Then the trajectory describes an exponential decay along the real eigenvectors and an inward spiral in the Re(*v*_*j*_), Im(*v*_*j*_) plane that is parametrised by *c*_*j*_, and given by Re(exp(*λ*_*j*_*t*)*c*_*j*_*v*_*j*_). We then obtain an upper bound for the deviation of the trajectory starting at *x*(0) in a direction *y*_*k*_ by maximising the contribution of each eigenvector separately.

Now, setting *y*_*kj*_ : = *y*_*k*_ ⋅ *v*_*j*_ for *v*_*j*_ real, and *y*_*kj*_ : = |*y*_*k*_ ⋅ *v*_*j*_| for *v*_*j*_ complex, this leads to the estimate:


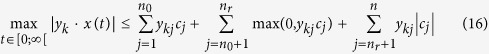


where the first sum is over real eigenvectors corresponding to null eigenvalues, the second is over nonzero real eigenvectors and the last is over the complex eigenvectors.

Setting the right hand side of Eq. 16 smaller than 1 defines a region *V*_*c*_ in 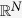 spanned by the real and imaginary parts of the coefficients *c*_*j*_. This region is mapped to the state space by 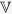 and thus its volume is related to the corresponding region in phase space by a determinant factor. As it is defined by a weaker inequality than 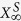 it follows that


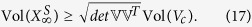


The inequalities Eq. 16 together with the matrix 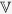 can be used to efficiently estimate the total survivability as well as the conditional survivability. Remarkably, for systems with a purely imaginary spectrum, the bounds of Eqs 16 and 17 hold with equality.

In the SI we also derive a lower bound for Vol(*V*_*c*_).

This lower bound demonstrates that for the survivability of a linear system, the eigenvectors play a crucial role. In fact, the eigenvalues do not enter the bound at all, except in terms of classifying the corresponding eigenvectors in separate classes. This demonstrates that the survivability captures substantially different information about the linear system than eigenvalue-based stability measures like relaxation time, or the master stability function.

### Relationship to Similar Concepts

Survivability is related to a number of concepts in other fields, notably control theory. From this perspective it can be seen as a so far unstudied, simplifying case where a number of distinct concepts from various fields intersect. In this section we discuss a number of such concepts and their precise relationship to survivability.

Survivability is conceptually similar to the notion of finite time stability as studied for linear control systems^13^,^14^. There the focus is on finding a particular control scheme that will ensure that the resulting closed loop system stays within a particular region for some time, possibly in the presence of perturbations of the dynamical equations. From our perspective this can be seen as attempting to find systems with *S*(*t*) = 1. As the focus there is on perturbed dynamics in linear control systems, the actual overlap of methods is very small, in particular it is not possible to extend the methods to high-dimensional non-linear systems.

Another concept from control theory which is similar to the basin of survival is the viability kernel defined by Aubin *et al.* in the context of viability theory^37^,^38^. They introduce the notion of an environment *K* that contains all desirable states. Within the environment, there is the so-called viability kernel *V*^39^,^40^ as the set of all initial conditions from which the system *can* stay within the environment. This basically is a more general version of our infinite-time basin of survival for non-deterministic systems or systems with multiple evolution paths and a management process. Consequently, *K*\*V* corresponds to the set of finite-time surviving states in deterministic systems. The viability kernel’s volume is proposed as a measure of the degree of viability^38^, in the limit of no control it thus reduces to our total survivability. However, we are not aware of this special case ever being considered in the context of viability theory. Whereas survivability measures the ability of the intrinsic dynamics to withstand perturbation, viability theory is concerned with the question of the power of control. Beyond this conceptual difference, evaluating survivability also requires very different technical methods, analytically as well as numerically. As far as we are aware, sampling based methods, which are efficient and natural for survivability, are impossible for viability. This is due to the fact that whether a particular point belongs to the viable set depends on the optimal control, which might not be known.

There are two concepts that share some formal similarity to survivability in the context of deterministic systems, transient times and open systems.

The study of transient life times^15^,^19^,^41^,^42^ is only related to the survivability in the non-typical special case that the attractor (or a small epsilon environment around it) is the only undesirable region. In our example of integrate-and-fire neurons this is the case, but in the power grid there is no clear relationship between the strength of the transient (which might kill the system) and the return time to the attractor. In fact, there, the attractor we start from is in the desirable region. Transients life times are a special case, and not a typical one, of survivability. The latter is far more general, going beyond the focus on the length of transients and their distribution, and typically captures genuinely different information of the system (e.g. the linear analysis mainly depends on eigenvectors, not eigenvalues).

The theory of open systems, on the other hand, is generally concerned with ergodic systems. For leaky chaotic systems^43^ the asymptotic behaviour of the survival probability is the key observable. At the formal level there is an analogy to our definitions, however, the total survivability, the size of the total phase space that leaks, is never considered as an observable in the literature. Indeed it is often the case that it is the whole phase space. Nor is the cumulative leakage ever interpreted as a stability measure or are efficient methods to estimate it for high-dimensional systems being discussed. In fact, as in the case of transient times, leaky systems can be seen as a special case of our discussion. Specifically it is the conditional survivability with the conditional space chosen as the space of surviving states *X*^*S*^.

The closest analogy to our deterministic survivability is simply the survival analysis in the the context of stochastic systems. The concept of the so-called first hitting time and survival probability^44^,^45^,^46^, which can be studied for the case of stochastic perturbations to deterministic systems by quasi-potentials^47^,^48^,^49^, map directly to our work. The first hitting time *t* measures when a system is expected to first hit the forbidden region *X*^−^. The cumulative of the probability of first hitting the undesirable region before *t* is then 1 − *S*(*t*). Our definitions given above can be seen as a deterministic version of these concepts. The role of stochasticity in the evolution is replaced by a probabilistic initial perturbation. Here similar sampling based methods are possible and necessary. The type of semi-analytic analysis we performed for the linear case would however be hard to duplicate. From this perspective what we have demonstrated is how to successfully apply methods and concepts from stochastic systems in the study of their deterministic counterparts.

The key insight in our work, as it is for *S*_*B*_, is that restricting ourselves to probabilistic notions enables a considerably wider applicability of our analysis, as well as new numerical and analytic methods. Put differently, by asking not about the geometry of sets in phase space but merely about their volume, we can access high-dimensional non-linear systems that are out of reach for detailed geometric analysis. The challenge then lies in defining interesting sets that capture concepts of interest. As such we take it as a confirmation for the wide interest of the specific sets that survivability is based on, that it occurs a the intersection of a number of well studied concepts.

## Additional Information

**How to cite this article**: Hellmann, F. *et al.* Survivability of Deterministic Dynamical Systems. *Sci. Rep.* **6**, 29654; doi: 10.1038/srep29654 (2016).

## Supplementary Material

Supplementary Information

## Figures and Tables

**Figure 1:**
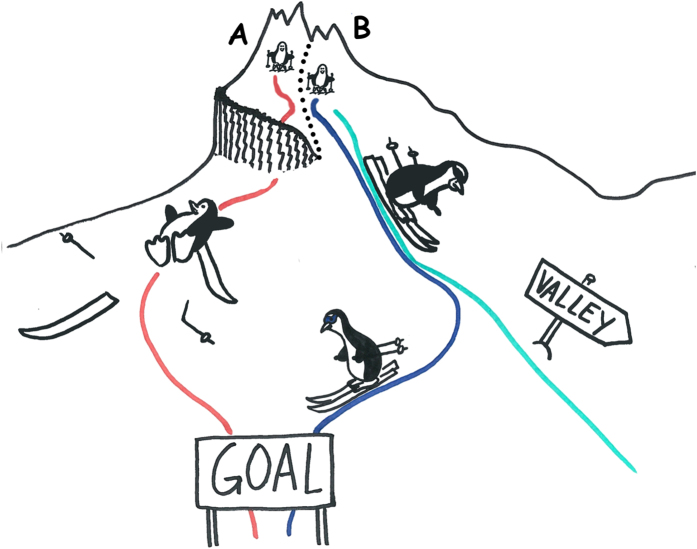
Survivability cartoon. A penguin can ski down the mountain starting anywhere on the slope. Starting at A the penguin will tumble over the cliffs, passing an undesirable state although ultimately reaching the goal. Starting at B the penguin will reach the goal standing on its feet. Starting even further to the right, it might end up in the valley, which might or might not be desirable.
