# Supplementary material for: Survivability of Deterministic Dynamical Systems
Source: Sci Rep. 2016 Jul 13;6:29654. doi: 10.1038/srep29654 (PMC4942794; doi:10.1038/srep29654)
Supplement: Supplementary Information [file srep29654-s1.pdf]

Survivability of Deterministic Dynamical Systems

Frank Hellmann[1,*,+], **Paul Schultz**[1,2,*,+], **Carsten Grabow**[1], **Jobst Heitzig**[1], **and Jürgen Kurths**[1,2,3,4]

[1]Potsdam Institute for Climate Impact Research, P.O. Box 60 12 03, 14412 Potsdam, Germany
[2]Department of Physics, Humboldt University of Berlin, Newtonstr. 15, 12489 Berlin, Germany
[3]Institute for Complex Systems and Mathematical Biology, University of Aberdeen, Aberdeen AB24 3UE, United Kingdom
[4]Department of Control Theory, Nizhny Novgorod State University, Gagarin Avenue 23, 606950 Nizhny Novgorod, Russia
[*]hellmann||pschultz @pik-potsdam.de
[+]These authors contributed equally to the research presented.

ABSTRACT

Supplementary information; extending the mathematical description of survivabilty and deriving (semi-)analytic bounds.

Contents

1 Formal Definition and Derivation of Analytic Bounds **1**
1.1 Formal definition and basic properties . . . 1
1.2 Conditional survivability . . . 2
1.3 Linear Systems . . . 2
1.4 Upper bound on the deviation of a single trajectory. . . . 3
1.5 A lower bound for the total survivability . . . 3
1.6 The case of vanishing real parts. . . . 4
1.7 The purely imaginary case. . . . 4
2 Relationship to Basin Stability **5**
3 Pulse-Coupled Integrate-and-Fire Oscillators **6**
References **6**

1 Formal Definition and Derivation of Analytic Bounds

As noted in the main text, the survivability of a linear system is amenable to analytic study. In this appendix we will give a more mathematically precise definition of survivability and then a detailed derivation of the results used in the main body of the text as well as some closely related ones.

1.1 Formal definition and basic properties

Consider a dynamical system with states x in a state space X giving rise to trajectories $x(t)$ under some evolution map $\sigma(t)$. Now we define a desirable region $X^+ \subset X$ with its complement $X^- = X \setminus X^+$, where the former contains all states x that are stated to be desirable. In the penguin example (Fig. 1) X^- would contain the cliff and the valley. In the context of Earth System science, such a desirable region has variously been called the *safe operating space* within *planetary boundaries*[1] or the *sunny region*.[2]

According to the main text, we define the finite-time survivability $S(t)$ of the dynamical system at time t to be the fraction of trajectories starting in X^+ that stay within X^+ for the entire duration $[0,t]$. Put another way, if entering the region X^- terminates the system, $S(t)$ is the fraction of trajectories starting in X^+ still alive after time t. We call the part of X^+ from which trajectories start that stay alive at least for time t the *finite-time* or t*-time basin of survival* X_t^S. We then have

$$S(t) := S_{\mu^+}(t) = \frac{\mu(X_t^S)}{\mu(X^+)}, \tag{1}$$

where μ is an inner measure on X determining the volume of the sets X_t^S and X^+ in the phase space. By construction, $S_{\mu^+}(t)$ takes values on the unit interval.

We define the total survivability $S_{\mu^+}(t \to \infty)$ as the limit

$$S_\infty := \lim_{t\to\infty} S(t)\,. \tag{2}$$

Each t-time basin of survival is a subset of the previous ones $X_t^S \supset X_{t'}^S$ (for $t' > t$), as trajectories returning to X^+ after leaving it once do not contribute to $X_{t'}^S$. Hence, $S(t)$ is monotonically decreasing and bounded by 0 from below, therefore the limit in Eqn. 2 exists. The use of an inner measure here avoids subtleties involving non-measurable sets, like fractal[3,4] or riddled[5,6] basins of attraction.

Note, however, that if X^+ is an open set, and the map $\sigma(t) : X \to X$ is continuous for all t, then the images of X^+ under $\sigma(t)^{-1}$ are also open. As $X_t^S = \bigcup_{0<t'<t} \sigma(t')^{-1} X^+$ is a union of open sets, it is itself open and therefore measurable if μ is a Borel measure. Thus in this important special case, which covers all applications we are considering in the main text, no such subtleties exist.

In some applications, the choice for the set X^+ might have an infinite volume, even though X_t^S becomes finite for sufficiently large t. In that case one can still consider the unnormalised measure $\mu(X_t^S)$ as a relative measure of the survivability of a system.

1.2 Conditional survivability

The conditional survivability $S^C(t)$ measures the response of the system to restricted perturbations. For example, we might be interested in the survivability, given perturbations that are localised at a node in a network. Given a subset of the state space $C \subset X$, we define the conditional survivability as the fraction of trajectories starting in $X^+ \cap C$ that stay in X^+. That is

$$S^C(t) = \frac{\mu|_C(X_t^S \cap C)}{\mu|_C(X^+ \cap C)}\,, \tag{3}$$

where $\mu|_C$ is an inner measure on the smallest sub-manifold containing C. In the case of a network and perturbations at a single node, the phase space typically is the product of phase spaces at the nodes, and the volume measure likewise factorises, thus there are natural choices for C and $\mu|_C$.

1.3 Linear Systems

We consider the case of a linear dynamic in $X = \mathbb{R}^N$, the standard Lebesgue measure $\mathrm{Vol}(X) = \int_X \mathrm{d}x^N$ and a polyhedral sunny region given by m linear conditions $y_k \cdot x(t) < 1$ for a set of vectors y_k, $k = 1\ldots m$ in $\mathbb{R}^N$.

The dynamics is given by a linear system of ordinary differential equations

$$\dot{x}(t) = Lx(t) \tag{4}$$

with $x \in X = \mathbb{R}^N$ and $L \in \mathbb{R}^{N\times N}$. In general, L has a complex spectrum, and we assume that all eigenvalues have non-positive real part. We denote the number of real eigenvalues of L as n_r, the number of pairs of complex conjugate complex eigenvalues n_c. Then we have a total of $n = n_r + n_c$ independent eigenvalues and $n_r + 2n_c = N$. We denote the eigenvalues as λ_i and $\overline{\lambda_i}$, and the corresponding eigenvectors as v_i and $\overline{v_i}$ respectively.

Assuming that the real and imaginary parts of the eigenvectors of L span the entire space X, the general solution to the dynamical equations are then given by the matrix exponential

$$\begin{aligned} x(t) &= \mathrm{e}^{Lt}x(0) \\ &= \sum_{j=1}^{n} \mathsf{Re}\left(c_j \mathrm{e}^{\lambda_j t} v_j\right) \\ &= \sum_{j=1}^{n_r} c_j \mathrm{e}^{\lambda_j t} v_j + \sum_{j=n_r+1}^{n} \mathsf{Re}\left(c_j \mathrm{e}^{\lambda_j t} v_j\right), \end{aligned} \tag{5}$$

where the coefficients c_j are real for $j \le n_r$ and complex above. To determine them we introduce a convenience map ι as follows:

$$\iota : \mathbb{R}^{n_r} \otimes \mathbb{C}^{n_c} \to \mathbb{R}^N$$
$$\iota(c)_j = \begin{cases} c_j & \text{if } j \leq n_r \\ \mathsf{Re}\,(c_j) & \text{if } n_r < j \leq n_c \\ -\mathsf{Im}\left(c_{(j-n_c)}\right) & \text{if } n_c < j\ . \end{cases} \tag{6}$$

This is a real-linear map. Then we can define the real matrix $\mathbb{V}$ as:

$$\begin{aligned} \mathbb{V} = &[v_1, \ldots, v_{n_r}, \\ &\mathsf{Re}\,(v_{n_r+1}), \ldots, \mathsf{Re}\,(v_n), \\ &-\mathsf{Im}\,(v_{n_r+1}), \ldots, -\mathsf{Im}\,(v_n)]. \end{aligned} \tag{7}$$

and obtain

$$x(0) = (\mathbb{V} \circ \iota)(c)\ , \tag{8}$$

which can be readily inverted as we assumed $\mathbb{V}$ to have full rank.

1.4 Upper bound on the deviation of a single trajectory.

The inner product of $x(t)$ with a boundary vector $y \in \mathbb{R}^N$ is then simply given by:

$$y \cdot x(t) = \sum_{j=1}^{n_r} c_j \mathrm{e}^{\lambda_j t} y \cdot v_j + \sum_{j=n_r+1}^{n_r+n_c} \mathsf{Re}\left(c_j \mathrm{e}^{\lambda_j t} y \cdot v_j\right) \tag{9}$$

Now, to obtain an upper bound on this for all times we can maximise each individual contribution. As the eigenvalues have non-positive real part each complex contribution has magnitude of at most $|c_j y \cdot v_j|$. The maximum of the real contribution depends on the sign of $c_j y \cdot v_j$ and is given by

$$\max(0, c_j y \cdot v_j) \tag{10}$$

unless $\lambda_j = 0$ in which case the contribution is exactly equal $c_j y \cdot v_j$. While this does not happen generically it occurs due to symmetries in the system, and thus we will treat it separately. We denote the number of zero eigenvalues by n_0. We have:

$$\begin{aligned} \max_{t \in [0;\infty[} |y \cdot x(t)| \leq &\sum_{j=1}^{n_0} c_j y \cdot v_j \\ &+ \sum_{j=n_0+1}^{n_r} \max(0, c_j y \cdot v_j) \\ &+ \sum_{j=n_r+1}^{n} |c_j y \cdot v_j|\ . \end{aligned} \tag{11}$$

This is the key approximation for our analysis. In the next section we will use this estimate to give a lower bound for the total survivability, afterwards we will show when this bound becomes tight.

1.5 A lower bound for the total survivability

For a boundary vector y_k let us define $y_{kj} = y_k \cdot v_j$ for $j \leq n_r$ and $y_{kj} = |y_k \cdot v_j|$ for $n_r < j \leq n$. Then the inequalities

$$\sum_{j=1}^{n_0} y_{kj} c_j + \sum_{j=n_0+1}^{n_r} \max(0, y_{kj} c_j) + \sum_{j=n_r+1}^{n} y_{kj} |c_j| < 1 \tag{12}$$

define a region V_c in $\mathbb{R}^{n_r} \otimes \mathbb{C}^{n_c}$ that is mapped to a subset of X_∞^S by the linear transformation $\mathbb{V} \circ \iota$. This is a subset of X_∞^S as the initial conditions in this region have an inner product with the boundary vectors y_k bounded by Eqn. 11. Thus, taking the effect of the transformation $\mathbb{V}$ into account, we have the lower bound

$$\mathrm{Vol}(X_\infty^S) \geq \sqrt{\det \mathbb{V}\mathbb{V}^T}\,\mathrm{Vol}(V_c)\ . \tag{13}$$

This bound can be evaluated numerically quite easily. All that is needed is to sample X^+ and take the fraction of samples for which the image under the linear map $(\mathbb{V} \circ \iota)^{-1}$ satisfies Eqn. 12. This also makes it very easy to numerically estimate the lower bound of conditional survivability, by sampling from $C \cap X^+$ instead. The data for the semi-analytic figures in the power grid section of the results was computed in this way.

In order to proceed with the analytic calculations we have to consider further special cases. First we will show that the bound Eqn. 13 is actually exact for some cases.

1.6 The case of vanishing real parts.

Let us now consider the case where the real part of all λ_i is zero, and $\frac{\mathrm{Im}(\lambda_i)}{\mathrm{Im}(\lambda_j)}$ is irrational. Thus $n_0 = n_r$. In that case the trajectory with initial conditions $x(0) = \mathbb{V} \circ \iota \circ c$ is dense on the torus:

$$\mathbb{T} = \left\{ \sum_{j=1}^{n_r} c_j v_j + \mathrm{Re}\left(\sum_{j=n_r+1}^{n} c_j e^{i\phi_j} v_j \right) \middle| \phi_j \in [0; 2\pi[\right\} \tag{14}$$

The maximum of the torus along the y direction is obtained exactly by maximising each contribution independently, which means tuning the ϕ_i so that $c_j e^{i\phi_j} \hat{x} \cdot v_j$ are real, thus the real part equals the absolute value:

$$\max_{\phi_i \in [0;2\pi[} |y \cdot \mathbb{T}| \leq \sum_{j=1}^{n_r} c_j y \cdot v_j + \sum_{j=n_r+1}^{n} |c_j y \cdot v_j| \tag{15}$$

with equality if $\frac{\mathrm{Im}(\lambda_i)}{\mathrm{Im}(\lambda_j)}$ is irrational. Therefore, in this case, the general bound Eqn. 11 holds with equality. We have:

$$\max_{t \in [0;\infty[} |y \cdot x(t)| = \sum_{j=1}^{n_r} c_j(x_0) y \cdot v_j + \sum_{j=n_r+1}^{n} |c_j(x_0)||y \cdot v_j| \tag{16}$$

and therefore also

$$\mathrm{Vol}(X_\infty^S) = \sqrt{\det \mathbb{V}\mathbb{V}^T}\,\mathrm{Vol}(V_c)\ . \tag{17}$$

1.7 The purely imaginary case.

For the case $n_r = n_0 = 0$ we can give an explicit lower bound for $\mathrm{Vol}(V_c)$. To do so, define $\tilde{y}_j = \min_k y_{kj}$. The volume of the space $\tilde{V}_c$ defined by the inequality

$$\sum_{j=1}^{n} \tilde{y}_j |c_j| < 1 \tag{18}$$

is a lower bound for the volume of V_c. The two spaces have the same volume if there is one condition that dominates all others, that is, if there is a k' such that $\tilde{y}_j = y_{k'j}$ for all j. This means that any trajectory that leaves the allowed region also crosses the boundary defined by $x \cdot y_k \leq 1$.

We now need to evaluate the $2n = 2n_c = N$ dimensional integral

$$\mathrm{Vol}(\tilde{V}_c) = \int_{\tilde{V}_c} \prod_{j=1}^{n} \mathrm{d}c_j^r \mathrm{d}c_j^i\ , \tag{19}$$

with $c_j = c^r + ic^i_j$. We begin by changing $\mathrm{d}c^r_j\mathrm{d}c^i_j$ to polar coordinates $r_j\mathrm{d}r_j\mathrm{d}\phi_j$ and rescaling:

$$\begin{aligned}\mathrm{Vol}(\tilde{V}_c) &= \left(\prod_j \tilde{y}_j^2\right)\int_{\sum_j r_j<1}\prod_{j=1}^n r_j\mathrm{d}r_j\mathrm{d}\phi_j \\ &= (2\pi)^n\int_{\sum_j r_j<1}\prod_{j=1}^n r_j\mathrm{d}r_j\,. \end{aligned}\tag{20}$$

The integral can now be written as

$$\mathrm{Vol}(\tilde{V}_c) = \left(\prod_j \tilde{y}_j^2\right)(2\pi)^n\int_0^{1-\sum_{j>1}r_j} r_1\mathrm{d}r_1\int_0^{1-\sum_{j>2}r_j} r_2\mathrm{d}r_2\int_0^{1-\sum_{j>3}r_j} r_3\mathrm{d}r_3\ldots\,. \tag{21}$$

This can be calculated by beta functions[1]:

$$\mathrm{Vol}(\tilde{V}_c) = \frac{(2\pi)^n}{(2n+1)!}\prod_j \tilde{y}_j^2\,. \tag{22}$$

This finally means that we obtain the lower bound on the region of total survivability of a linear system with no real eigenvalues and all real parts of the eigenvalues equal to zero of

$$\mathrm{Vol}(X^S_\infty) \geq \frac{(2\pi)^n}{(2n+1)!}\sqrt{\det \mathbb{V}\mathbb{V}^T}\prod_j \tilde{y}_j^2\,. \tag{23}$$

2 Relationship to Basin Stability

Let us further consider the relationship between basin stability and survivability. Consider the union of all attractors A in X. Following the terminology of,[2] we split the set A into desirable attractors A^+ and undesirable attractors A^-. Define X_A^+ and X_A^- to be the basin of attraction of A^+ and A^- respectively. The basin stability of A^+ with respect to some initial region X^0 is then defined as

$$S_B = \frac{\mathrm{Vol}(X^0\cap X_A^+)}{\mathrm{Vol}(X^0)}\,. \tag{24}$$

Note, that defining basin stability requires knowledge of the respective attractor. Efficiently evaluating it numerically requires a criterion to evaluate whether the system will converge to a certain attractor.

Let us assume that the sunny region cleanly separates the set of attractors, that is, there are some attractors that are entirely sunny and others that are entirely shaded, but none that intersect both regions. In this case, we can establish a quantitative relationship between basin stability and survivability. We can choose $A^+ = A\cap X^+$ (i.e. desirable attractors are contained inside the sunny region), and we know that asymptotically the trajectories converge either to A^- or A^+ or diverge. Thus every trajectory that does not contribute to the basin stability also has to leave the sunny region eventually and can not contribute to the survivability either. Thus if

$$\begin{aligned} & A^+ = A\cap X^+ \\ \text{and}\quad & X^0 = X^+\,, \\ \text{then}\quad & S_B \geq S_\infty\,. \end{aligned}\tag{25}$$

The difference between the two values is exactly the measure of initial conditions whose trajectories leave the sunny region intermittently but eventually return to it and stay. Thus we see that whether basin stability or survivability is the appropriate measure depends on whether the forbidden region is merely unpleasant, and we want our stay there to be finite, or whether the forbidden region is deadly and we absolutely do not want the system to enter it at all.

[1] The detailed calculation can be found here http://math.stackexchange.com/a/207605. (Accessed: May 31, 2016)

3 Pulse-Coupled Integrate-and-Fire Oscillators

We here give further details about the integrate-and-fire model for coupled neurons we used in the main text. Firstly, the free dynamics of an oscillator j is given by

$$\dot{\phi}_j(t) = 1 \,. \tag{26}$$

When an oscillator j reaches the threshold, $\phi_j(t) = 1$, its phase is reset to zero, $\phi_j(t^+) = 0$, and the oscillator emits a pulse that is sent to all oscillators i possessing an in-link from j. After a delay time τ this pulse induces a phase jump in the receiving oscillator i according to

$$\phi_i((t+\tau)^+) := \min\left(1, \frac{\mathrm{e}^{b\varepsilon_{ij}}-1}{\mathrm{e}^b-1} + \mathrm{e}^{b\varepsilon_{ij}}\phi_i(t+\tau)\right) \tag{27}$$

The phase dependence is determined by a twice continuously differentiable function $U(\phi)$ that is assumed to be strictly increasing, $U'(\phi) > 0$, concave (down), $U''(\phi) < 0$, and normalised such that $U(0) = 0$ and $U(1) = 1$.

This model, originally introduced by Mirollo and Strogatz,[7] is equivalent to different well known models of interacting threshold elements if $U(\phi)$ is chosen appropriately. Here we take functions of the form

$$U_b(\phi) = b^{-1}\ln(1+(e^b-1)\phi), \tag{28}$$

where $b > 0$ parametrises the curvature of U, that determines the strength of the dissipation of individual oscillators. The function U approaches the linear, non-leaky case in the limit $\lim_{b\to 0} U_b(\phi) = \phi$. Other nonlinear choices of $U \neq U_b$ give results similar to those reported above.

The considered graphs are strongly connected, i.e. there exists a directed path between any pair of nodes. We normalise the total input to each node $\sum_{j=1}^{N} \varepsilon_{ij} = \varepsilon$ such that the fully synchronous state exists. Furthermore for any node i all its k_i incoming links have the same strength $\varepsilon_{ij} = \varepsilon/k_i$.

References

1. Rockström, J. *et al.* A safe operating space for humanity. *Nature* **461**, 472–475 (2009). 461472a.
2. Heitzig, J., Kittel, T., Donges, J. F. & Molkenthin, N. Topology of sustainable management of dynamical systems with desirable states: from defining planetary boundaries to safe operating spaces in the Earth system. *Earth System Dynamics* **7**, 21–50 (2016).
3. Nusse, H. E. & Yorke, J. A. Wada basin boundaries and basin cells. *Physica D: Nonlinear Phenomena* **90**, 242–261 (1996).
4. Nusse, H. E. & Yorke, J. A. Basins of attraction. *Science* **271**, 1376–1380 (1996).
5. Alexander, J., Yorke, J., You, Z. & Kan, I. Riddled basins. *International Journal of Bifurcation and Chaos* **2**, 795–813 (1992).
6. Lai, Y. & Grebogi, C. Intermingled basins and two-state on-off intermittency. *Physical Review E* **52**, R3313–R3316 (1995).
7. Mirollo, R. & Strogatz, S. Synchronization of pulse-coupled biological oscillators. *Siam Journal on Applied Mathematics* **50**, 366 (1990).
